# {5,5′-Dihydroxy-2,2′-[(2-hydroxypropane-1,3-diyl)bis(nitrilomethanylyli­dene)]diphenolato}nickel(II) dihydrate

**DOI:** 10.1107/S1600536813026007

**Published:** 2013-09-25

**Authors:** Amitabha Datta, Jui-Hsien Huang, Shiann-Cherng Sheu

**Affiliations:** a Department of Chemistry, National Changhua University of Education, Changhua, 50058, Taiwan; b Department of Occupational Health and Safety, Chang Jung Christian University, Tainan City, 71101, Taiwan

## Abstract

In the title complex, [Ni(C_17_H_16_N_2_O_5_)]·2H_2_O, the Ni^II^ ion is four-coordinated by two azomethine N and two phenolato O atoms of the tetradentate Schiff base ligand in a slightly distorted square-planar geometry. In the six-membered ring containing the metal, the azomethine N atoms and the three C atoms of the connecting 1,3-di­amino­propane-2-ol, all atoms except the metal are disordered over two sets of sites with an occupacy ratio of 0.566 (3):0.434 (3). The central C atom of the major component is significantly out of the mean plane of the remaing atoms while the conformation of this ring in the minor component is noticeably different. In the crystal, O—H⋯O hydrogen bonds involving the lattice water mol­ecules and the hy­droxy groups are observed.

## Related literature

For related structures, see: Averseng *et al.* (2001 ▶); Donmez *et al.* (2007 ▶). For ring-puckering analysis, see: Cremer & Pople (1975 ▶). 
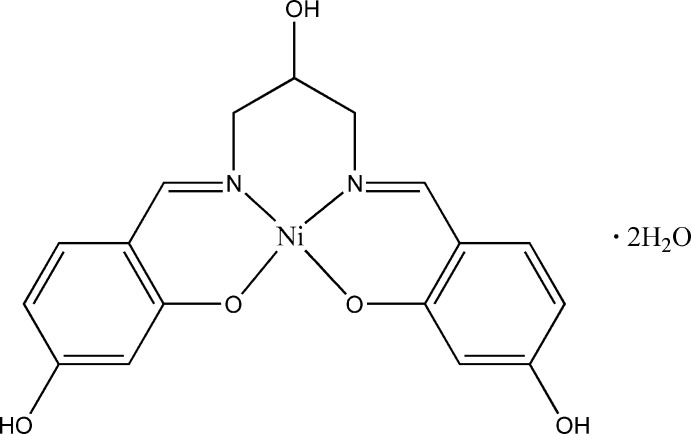


## Experimental

### 

#### Crystal data

[Ni(C_17_H_16_N_2_O_5_)]·2H_2_O
*M* *_r_* = 423.06
Monoclinic, 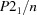

*a* = 8.201 (1) Å
*b* = 17.887 (3) Å
*c* = 11.863 (2) Å
β = 92.444 (3)°
*V* = 1738.6 (5) Å^3^
*Z* = 4
Mo *K*α radiation
μ = 1.16 mm^−1^
*T* = 293 K
0.13 × 0.10 × 0.06 mm

#### Data collection

Bruker BREEZE CCD area-detector diffractometer
Absorption correction: multi-scan (*SADABS*; Bruker, 2008 ▶) *T*_min_ = 0.599, *T*_max_ = 0.746
23452 measured reflections
4164 independent reflections
2986 reflections with *I* > 2σ(*I*)
*R*_int_ = 0.046

#### Refinement

*R*[*F* ^2^ > 2σ(*F* ^2^)] = 0.037
*wR*(*F* ^2^) = 0.093
*S* = 1.04
4164 reflections
263 parameters
18 restraints
H-atom parameters constrained
Δρ_max_ = 0.32 e Å^−3^
Δρ_min_ = −0.35 e Å^−3^

### 

Data collection: *APEX2* (Bruker, 2007 ▶); cell refinement: *SAINT* (Bruker, 2007 ▶); data reduction: *SAINT*; program(s) used to solve structure: *SHELXS97* (Sheldrick, 2008 ▶); program(s) used to refine structure: *SHELXL97* (Sheldrick, 2008 ▶); molecular graphics: *SHELXP* (Sheldrick, 2008 ▶); software used to prepare material for publication: *publCIF* (Westrip, 2010 ▶).

## Supplementary Material

Crystal structure: contains datablock(s) I, New_Global_Publ_Block. DOI: 10.1107/S1600536813026007/mw2116sup1.cif

Structure factors: contains datablock(s) I. DOI: 10.1107/S1600536813026007/mw2116Isup2.hkl

Additional supplementary materials:  crystallographic information; 3D view; checkCIF report

## Figures and Tables

**Table 1:** Hydrogen-bond geometry (Å, °)

*D*—H⋯*A*	*D*—H	H⋯*A*	*D*⋯*A*	*D*—H⋯*A*
O1*W*—H1*W*⋯O2	0.82	1.96	2.770 (2)	167
O1*W*—H1*W*⋯O1	0.82	2.53	3.087 (2)	127
O1*W*—H2*W*⋯O4^i^	0.82	2.18	2.951 (3)	158
O2*W*—H3*W*⋯O1^ii^	0.82	1.98	2.787 (3)	168
O2*W*—H4*W*⋯O3^iii^	0.82	2.04	2.837 (2)	165
O3—H3*O*⋯O1*W* ^iv^	0.82	1.87	2.665 (3)	165
O4—H4*O*⋯O2*W* ^v^	0.82	1.81	2.626 (3)	171
O5—H5*OA*⋯O1*W* ^vi^	0.82	1.97	2.78 (3)	166
O5′—H5*OB*⋯O1*W* ^vi^	0.82	2.28	3.03 (4)	151

Symmetry codes: (i) 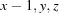; (ii) 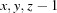; (iii) 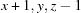; (iv) 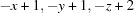; (v) 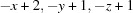; (vi) 
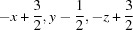
.

**Fig. 1.**
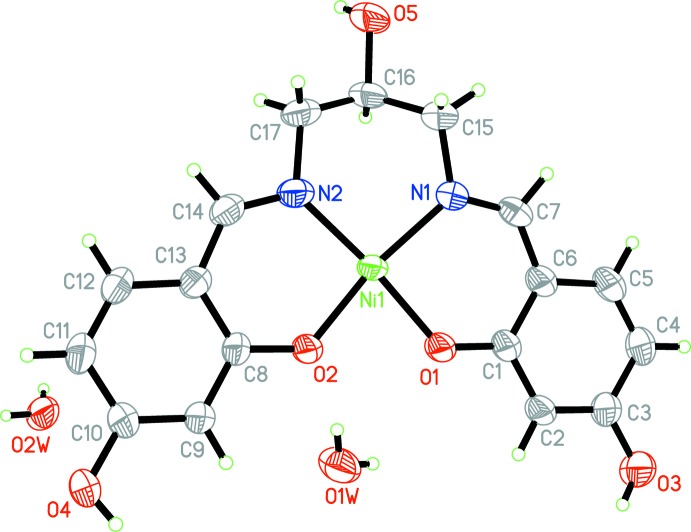
The molecular structure of the title complex, showing displacement ellipsoids at the 50% probability level.

**Fig. 2.**
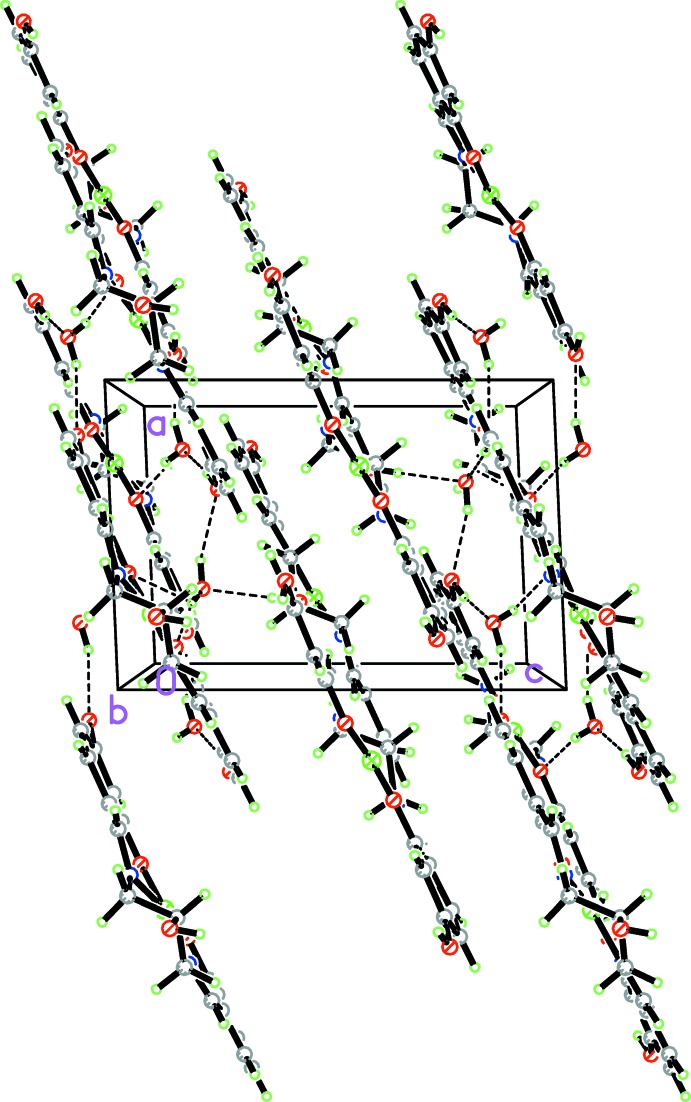
Packing diagram of the title compound as viewed down the b axis. Intermolecular O—H···O hydrogen bonds are shown as dashed lines.

## supplementary crystallographic information

### 1. Comment

In the title complex, [Ni(OC_6_H_3_OH)—CH=N—CH_2_—CH(OH)—CH_2_—N=CH- (OHC_6_H_3_O)], the Ni^II^ metal center is coordinated by a pair of phenolate O and imine N atoms from the corresponding Schiff base precursor (Fig. 1). The square-planar N_2_O_2_ coordination sphere is slightly distorted as indicated by the less than linear bond angles, N1—Ni1—O2 and N2—Ni1—O1 (170.11 (14)° and 172.70 (7)° respectively). In the coordination plane, the bond distances Ni1–N1, Ni1–N2, Ni1–O1 and Ni1–O2 are 1.980 (5), 1.971 (2), 1.926 (2) and 1.913 (2) Å, respectively. The dihedral angle between the two C1—C6 and C8—C13 benzene rings of the ligand is 4.95 (8)°. The atoms C7, N1, O5 and C15–C17 are disordered over two sites in a 57:43 ratio. A puckering analysis (Cremer & Pople, 1975) of the conformation of the major component of the six-membered ring containing the metal, the azomethine N atoms and the three carbon atoms of the connecting 1,3-diaminopropane-2-ol yielded the parameters Q = 0.579 (7) Å, θ = 110.6 (7)° and φ = 337.9 (8)° while for the minor component, the parameters are Q = 0.562 (8) Å, θ = 100.4 (10)° and φ = 44.6 (10)°. The N2—Ni1—N1 angle of the central chelating diamine portion is 95.06 (15)° while the O2—Ni1—O1 angle is 80.63 (7)° as a result of the steric congestion of the central six-membered chelate ring. In the crystal intermolecular O—H···O hydrogen bonds between water molecules and hydroxy groups are observed (Table 1 and Fig. 2).

### 2. Experimental

The Schiff base ligand, 4,4'-(1E,1'*E*)-(2-hydroxypropane-1,3-diyl)bis(azan-1-yl-1-ylidene) bis(methan-1-yl-1-ylidene)dibenzene-1,3-diol was prepared by condensation of 2,4-dihydroxybenzaldehyde (0.276 g, 2 mmol) and 1,3-diaminopropane-2-ol (0.090 g, 1 mmol) in methanol (25 mL). After 2 h reflux, the pale yellow solution was cooled to room temperature. The solvent was removed under reduced pressure and the Schiff-base ligand was obtained as a light-yellow liquid that was used without further purification. To prepare the complex, the Schiff base ligand (0.301 g, 1 mmol) was added to a methanolic solution (20 ml) of Ni(CH_3_COO)_2_· 4H_2_O (0.248 g, 1 mmol) which immediately produced an intensely brownish-red solution. The solution was heated to boiling and then kept undisturbed in a dark place. On cooling and after slow evaporation of the solution, dark brown plate-shaped single crystals of the complex separated out over 3 days. The crystals were filtered off and washed with water and dried in air.

### 3. Refinement

All of the H-atoms were placed in calculated positions (C—H 0.93 to 0.98 Å and O—H 0.82 Å) and were included in the refinement in the riding model approximation with *U*_iso_(H) = 1.2Ueq (C,*O*). The C7, N1, C15, C16, C17 and O5 atoms of the ligand are disordered over two sites with an occupancy ratio of 0.566 (3):0.434 (3). The SADI restraint and EADP constraint commands in the *SHELXL97* software were used for the disordered atoms.

### Crystal data

**Table:**

[Ni(C_17_H_16_N_2_O_5_)]·2H_2_O	*F*(000) = 880
*M**_r_* = 423.06	*D*_x_ = 1.616 Mg m^−^^3^
Monoclinic, *P*2_1_/*n*	Mo *K*α radiation, λ = 0.71069 Å
Hall symbol: -P 2yn	Cell parameters from 692 reflections
*a* = 8.201 (1) Å	θ = 2.1–27.8°
*b* = 17.887 (3) Å	µ = 1.16 mm^−^^1^
*c* = 11.863 (2) Å	*T* = 293 K
β = 92.444 (3)°	Thin plate, brown
*V* = 1738.6 (5) Å^3^	0.13 × 0.10 × 0.06 mm
*Z* = 4	

### Data collection

**Table:**

Bruker BREEZE CCD area-detector diffractometer	4164 independent reflections
Radiation source: fine-focus sealed tube	2986 reflections with *I* > 2σ(*I*)
Graphite monochromator	*R*_int_ = 0.046
ω–scan	θ_max_ = 28.0°, θ_min_ = 2.1°
Absorption correction: multi-scan (*SADABS*; Bruker, 2008)	*h* = −10→10
*T*_min_ = 0.599, *T*_max_ = 0.746	*k* = −23→23
23452 measured reflections	*l* = −15→15

### Refinement

**Table:**

Refinement on *F*^2^	Primary atom site location: structure-invariant direct methods
Least-squares matrix: full	Secondary atom site location: difference Fourier map
*R*[*F*^2^ > 2σ(*F*^2^)] = 0.037	Hydrogen site location: inferred from neighbouring sites
*wR*(*F*^2^) = 0.093	H-atom parameters constrained
*S* = 1.04	*w* = 1/[σ^2^(*F*_o_^2^) + (0.0388*P*)^2^ + 0.7206*P*] where *P* = (*F*_o_^2^ + 2*F*_c_^2^)/3
4164 reflections	(Δ/σ)_max_ < 0.001
263 parameters	Δρ_max_ = 0.32 e Å^−^^3^
18 restraints	Δρ_min_ = −0.35 e Å^−^^3^

### Special details

**Table:**

Geometry. All e.s.d.'s (except the e.s.d. in the dihedral angle between two l.s. planes) are estimated using the full covariance matrix. The cell e.s.d.'s are taken into account individually in the estimation of e.s.d.'s in distances, angles and torsion angles; correlations between e.s.d.'s in cell parameters are only used when they are defined by crystal symmetry. An approximate (isotropic) treatment of cell e.s.d.'s is used for estimating e.s.d.'s involving l.s. planes.
Refinement. Refinement of *F*^2^ against ALL reflections. The weighted *R*-factor *wR* and goodness of fit *S* are based on *F*^2^, conventional *R*-factors *R* are based on *F*, with *F* set to zero for negative *F*^2^. The threshold expression of *F*^2^ > σ(*F*^2^) is used only for calculating *R*-factors(gt) *etc*. and is not relevant to the choice of reflections for refinement. *R*-factors based on *F*^2^ are statistically about twice as large as those based on *F*, and *R*- factors based on ALL data will be even larger.

### Fractional atomic coordinates and isotropic or equivalent isotropic displacement parameters (Å^2^)

**Table:**

	*x*	*y*	*z*	*U*_iso_*/*U*_eq_	Occ. (<1)
Ni1	0.76757 (3)	0.236496 (16)	0.93756 (3)	0.03859 (11)	
N1	0.6337 (6)	0.1583 (3)	1.0070 (4)	0.0460 (10)	0.566 (3)
N1'	0.5940 (8)	0.1599 (4)	0.9475 (5)	0.0460 (10)	0.434 (3)
N2	0.9310 (2)	0.16644 (11)	0.88144 (19)	0.0466 (5)	
O1	0.63064 (19)	0.31366 (9)	0.99584 (15)	0.0473 (4)	
O2	0.88647 (18)	0.32255 (8)	0.89186 (15)	0.0450 (4)	
O3	0.1513 (2)	0.43146 (10)	1.11744 (16)	0.0566 (5)	
H3O	0.2114	0.4680	1.1241	0.068*	
O4	1.3374 (2)	0.46782 (10)	0.78268 (19)	0.0716 (6)	
H4O	1.2827	0.5012	0.8098	0.086*	
O5	0.757 (6)	−0.0234 (7)	0.894 (3)	0.071 (5)	0.566 (3)
H5OA	0.7605	−0.0387	0.8291	0.085*	0.566 (3)
O5'	0.770 (8)	−0.0278 (9)	0.919 (4)	0.071 (5)	0.434 (3)
H5OB	0.8157	−0.0371	0.8607	0.085*	0.434 (3)
C1	0.4836 (3)	0.30825 (13)	1.0367 (2)	0.0394 (5)	
C2	0.3974 (3)	0.37316 (13)	1.0600 (2)	0.0402 (5)	
H2	0.4450	0.4196	1.0484	0.048*	
C3	0.2424 (3)	0.36936 (14)	1.1002 (2)	0.0426 (6)	
C4	0.1706 (3)	0.30072 (15)	1.1231 (2)	0.0514 (7)	
H4	0.0676	0.2985	1.1528	0.062*	
C5	0.2534 (3)	0.23743 (15)	1.1013 (2)	0.0522 (7)	
H5	0.2057	0.1916	1.1165	0.063*	
C6	0.4093 (3)	0.23866 (13)	1.0564 (2)	0.0445 (6)	
C7	0.4929 (10)	0.1699 (5)	1.0482 (6)	0.048 (2)	0.566 (3)
H7A	0.4402	0.1282	1.0758	0.057*	0.566 (3)
C7'	0.4642 (14)	0.1703 (6)	1.0048 (8)	0.048 (2)	0.434 (3)
H7B	0.3984	0.1285	1.0139	0.057*	0.434 (3)
C8	1.0308 (3)	0.32654 (13)	0.84706 (19)	0.0370 (5)	
C9	1.1033 (3)	0.39629 (13)	0.8323 (2)	0.0428 (6)	
H9	1.0478	0.4394	0.8516	0.051*	
C10	1.2565 (3)	0.40182 (14)	0.7892 (2)	0.0506 (7)	
C11	1.3385 (3)	0.33891 (16)	0.7536 (3)	0.0620 (8)	
H11	1.4395	0.3433	0.7215	0.074*	
C12	1.2684 (3)	0.27072 (15)	0.7665 (3)	0.0556 (7)	
H12	1.3227	0.2284	0.7423	0.067*	
C13	1.1161 (3)	0.26237 (13)	0.8153 (2)	0.0419 (5)	
C14	1.0606 (3)	0.18853 (14)	0.8326 (2)	0.0481 (6)	
H14	1.1261	0.1508	0.8053	0.058*	
C15	0.6836 (7)	0.0784 (2)	1.0058 (5)	0.0631 (12)	0.566 (3)
H15A	0.5904	0.0472	1.0213	0.076*	0.566 (3)
H15B	0.7669	0.0698	1.0649	0.076*	0.566 (3)
C15'	0.6172 (9)	0.0838 (3)	0.9034 (6)	0.0631 (12)	0.434 (3)
H15C	0.6092	0.0852	0.8216	0.076*	0.434 (3)
H15D	0.5304	0.0519	0.9287	0.076*	0.434 (3)
C16	0.7488 (11)	0.0567 (5)	0.8937 (5)	0.0492 (18)	0.566 (3)
H16A	0.6720	0.0731	0.8332	0.059*	0.566 (3)
C16'	0.7765 (14)	0.0510 (7)	0.9401 (9)	0.0492 (18)	0.434 (3)
H16B	0.7802	0.0553	1.0225	0.059*	0.434 (3)
C17	0.9129 (16)	0.0834 (5)	0.8700 (10)	0.053 (3)	0.566 (3)
H17A	0.9379	0.0689	0.7938	0.063*	0.566 (3)
H17B	0.9915	0.0592	0.9214	0.063*	0.566 (3)
C17'	0.927 (2)	0.0850 (7)	0.9064 (15)	0.053 (3)	0.434 (3)
H17C	0.9612	0.0589	0.8397	0.063*	0.434 (3)
H17D	1.0095	0.0754	0.9658	0.063*	0.434 (3)
O1W	0.6893 (2)	0.44265 (10)	0.82950 (17)	0.0640 (5)	
H1W	0.7360	0.4051	0.8540	0.077*	
H2W	0.5895	0.4409	0.8310	0.077*	
O2W	0.8076 (2)	0.41714 (11)	0.1272 (2)	0.0741 (6)	
H3W	0.7637	0.3886	0.0811	0.089*	
H4W	0.9035	0.4214	0.1113	0.089*	

### Atomic displacement parameters (Å^2^)

**Table:**

	*U*^11^	*U*^22^	*U*^33^	*U*^12^	*U*^13^	*U*^23^
Ni1	0.03398 (17)	0.02135 (15)	0.0616 (2)	−0.00177 (12)	0.01543 (13)	−0.00026 (13)
N1	0.056 (3)	0.0281 (12)	0.056 (3)	−0.0013 (15)	0.020 (2)	0.001 (2)
N1'	0.056 (3)	0.0281 (12)	0.056 (3)	−0.0013 (15)	0.020 (2)	0.001 (2)
N2	0.0432 (11)	0.0274 (10)	0.0698 (14)	−0.0003 (8)	0.0094 (10)	−0.0024 (9)
O1	0.0345 (8)	0.0292 (8)	0.0802 (12)	−0.0041 (7)	0.0240 (8)	0.0010 (8)
O2	0.0334 (8)	0.0285 (8)	0.0745 (12)	−0.0016 (6)	0.0203 (8)	−0.0002 (8)
O3	0.0338 (9)	0.0478 (11)	0.0899 (14)	0.0001 (8)	0.0208 (9)	−0.0065 (10)
O4	0.0518 (11)	0.0453 (11)	0.1213 (17)	−0.0134 (9)	0.0476 (11)	−0.0103 (11)
O5	0.107 (7)	0.0272 (16)	0.082 (12)	−0.009 (3)	0.038 (10)	−0.002 (3)
O5'	0.107 (7)	0.0272 (16)	0.082 (12)	−0.009 (3)	0.038 (10)	−0.002 (3)
C1	0.0337 (11)	0.0353 (12)	0.0499 (14)	−0.0051 (9)	0.0112 (10)	0.0020 (10)
C2	0.0330 (11)	0.0314 (12)	0.0572 (15)	−0.0045 (9)	0.0117 (10)	0.0029 (10)
C3	0.0336 (12)	0.0433 (14)	0.0518 (14)	−0.0010 (10)	0.0107 (10)	−0.0011 (11)
C4	0.0346 (13)	0.0548 (16)	0.0664 (17)	−0.0081 (11)	0.0206 (12)	−0.0001 (13)
C5	0.0441 (14)	0.0437 (14)	0.0702 (17)	−0.0157 (12)	0.0187 (12)	0.0039 (13)
C6	0.0392 (12)	0.0344 (12)	0.0609 (15)	−0.0061 (10)	0.0147 (11)	0.0037 (11)
C7	0.052 (3)	0.0304 (14)	0.062 (6)	−0.0118 (19)	0.016 (4)	0.003 (3)
C7'	0.052 (3)	0.0304 (14)	0.062 (6)	−0.0118 (19)	0.016 (4)	0.003 (3)
C8	0.0300 (11)	0.0360 (12)	0.0457 (13)	−0.0004 (9)	0.0103 (10)	−0.0028 (10)
C9	0.0364 (12)	0.0330 (12)	0.0602 (15)	−0.0012 (10)	0.0164 (11)	−0.0032 (11)
C10	0.0426 (14)	0.0403 (14)	0.0705 (18)	−0.0093 (11)	0.0218 (12)	−0.0063 (12)
C11	0.0458 (15)	0.0545 (17)	0.088 (2)	−0.0060 (13)	0.0344 (14)	−0.0147 (15)
C12	0.0460 (14)	0.0447 (15)	0.0782 (19)	0.0008 (12)	0.0267 (13)	−0.0157 (14)
C13	0.0365 (12)	0.0346 (12)	0.0556 (15)	−0.0002 (10)	0.0116 (10)	−0.0079 (11)
C14	0.0408 (13)	0.0344 (13)	0.0700 (17)	0.0026 (10)	0.0133 (12)	−0.0108 (12)
C15	0.086 (3)	0.0290 (18)	0.077 (3)	0.0026 (19)	0.035 (3)	0.002 (2)
C15'	0.086 (3)	0.0290 (18)	0.077 (3)	0.0026 (19)	0.035 (3)	0.002 (2)
C16	0.069 (4)	0.0227 (19)	0.057 (6)	−0.003 (2)	0.012 (4)	0.001 (4)
C16'	0.069 (4)	0.0227 (19)	0.057 (6)	−0.003 (2)	0.012 (4)	0.001 (4)
C17	0.058 (3)	0.0264 (13)	0.075 (8)	0.0046 (14)	0.011 (5)	−0.005 (3)
C17'	0.058 (3)	0.0264 (13)	0.075 (8)	0.0046 (14)	0.011 (5)	−0.005 (3)
O1W	0.0493 (11)	0.0433 (11)	0.0997 (16)	0.0012 (8)	0.0081 (10)	0.0162 (10)
O2W	0.0406 (10)	0.0589 (13)	0.1241 (19)	−0.0093 (9)	0.0197 (11)	−0.0314 (12)

### Geometric parameters (Å, º)

**Table:**

Ni1—O2	1.9129 (15)	C6—C7'	1.448 (10)
Ni1—O1	1.9262 (15)	C7—H7A	0.9300
Ni1—N2	1.9713 (19)	C7'—H7B	0.9300
Ni1—N1	1.980 (5)	C8—C9	1.396 (3)
Ni1—N1'	1.983 (7)	C8—C13	1.404 (3)
N1—C7	1.290 (8)	C9—C10	1.380 (3)
N1—C15	1.486 (6)	C9—H9	0.9300
N1'—C7'	1.300 (10)	C10—C11	1.386 (3)
N1'—C15'	1.474 (9)	C11—C12	1.360 (4)
N2—C14	1.294 (3)	C11—H11	0.9300
N2—C17'	1.487 (12)	C12—C13	1.407 (3)
N2—C17	1.498 (9)	C12—H12	0.9300
O1—C1	1.322 (3)	C13—C14	1.415 (3)
O2—C8	1.320 (2)	C14—H14	0.9300
O3—C3	1.359 (3)	C15—C16	1.505 (8)
O3—H3O	0.8200	C15—H15A	0.9700
O4—C10	1.358 (3)	C15—H15B	0.9700
O4—H4O	0.8200	C15'—C16'	1.481 (10)
O5—C16	1.433 (10)	C15'—H15C	0.9700
O5—H5OA	0.8200	C15'—H15D	0.9700
O5'—C16'	1.432 (13)	C16—C17	1.466 (9)
O5'—H5OB	0.8200	C16—H16A	0.9800
C1—C2	1.393 (3)	C16'—C17'	1.449 (12)
C1—C6	1.410 (3)	C16'—H16B	0.9800
C2—C3	1.378 (3)	C17—H17A	0.9700
C2—H2	0.9300	C17—H17B	0.9700
C3—C4	1.393 (3)	C17'—H17C	0.9700
C4—C5	1.350 (4)	C17'—H17D	0.9700
C4—H4	0.9300	O1W—H1W	0.8201
C5—C6	1.406 (3)	O1W—H2W	0.8200
C5—H5	0.9300	O2W—H3W	0.8200
C6—C7	1.413 (8)	O2W—H4W	0.8200
			
O2—Ni1—O1	80.63 (7)	C10—C9—H9	119.7
O2—Ni1—N2	93.08 (8)	C8—C9—H9	119.7
O1—Ni1—N2	172.70 (7)	O4—C10—C9	122.5 (2)
O2—Ni1—N1	170.11 (14)	O4—C10—C11	116.3 (2)
O1—Ni1—N1	90.86 (14)	C9—C10—C11	121.1 (2)
N2—Ni1—N1	95.06 (15)	C12—C11—C10	118.7 (2)
O2—Ni1—N1'	161.6 (2)	C12—C11—H11	120.6
O1—Ni1—N1'	92.43 (19)	C10—C11—H11	120.6
N2—Ni1—N1'	94.7 (2)	C11—C12—C13	121.9 (2)
C7—N1—C15	114.3 (5)	C11—C12—H12	119.0
C7—N1—Ni1	124.4 (5)	C13—C12—H12	119.0
C15—N1—Ni1	121.2 (3)	C8—C13—C12	119.0 (2)
C7'—N1'—C15'	116.0 (7)	C8—C13—C14	123.9 (2)
C7'—N1'—Ni1	122.6 (7)	C12—C13—C14	117.1 (2)
C15'—N1'—Ni1	120.9 (5)	N2—C14—C13	128.7 (2)
C14—N2—C17'	114.4 (7)	N2—C14—H14	115.6
C14—N2—C17	110.0 (5)	C13—C14—H14	115.6
C14—N2—Ni1	122.71 (16)	N1—C15—C16	111.4 (5)
C17'—N2—Ni1	122.4 (7)	N1—C15—H15A	109.3
C17—N2—Ni1	126.5 (5)	C16—C15—H15A	109.3
C1—O1—Ni1	129.33 (14)	N1—C15—H15B	109.3
C8—O2—Ni1	129.38 (14)	C16—C15—H15B	109.3
C3—O3—H3O	109.5	H15A—C15—H15B	108.0
C10—O4—H4O	109.5	N1'—C15'—C16'	112.8 (7)
C16—O5—H5OA	109.6	N1'—C15'—H15C	109.0
C16'—O5'—H5OB	109.2	C16'—C15'—H15C	109.0
O1—C1—C2	119.4 (2)	N1'—C15'—H15D	109.0
O1—C1—C6	122.2 (2)	C16'—C15'—H15D	109.0
C2—C1—C6	118.4 (2)	H15C—C15'—H15D	107.8
C3—C2—C1	120.7 (2)	O5—C16—C17	107 (2)
C3—C2—H2	119.6	O5—C16—C15	105.8 (15)
C1—C2—H2	119.6	C17—C16—C15	116.7 (8)
O3—C3—C2	122.2 (2)	O5—C16—H16A	109.2
O3—C3—C4	116.8 (2)	C17—C16—H16A	109.2
C2—C3—C4	121.0 (2)	C15—C16—H16A	109.2
C5—C4—C3	118.8 (2)	O5'—C16'—C17'	113 (3)
C5—C4—H4	120.6	O5'—C16'—C15'	108 (3)
C3—C4—H4	120.6	C17'—C16'—C15'	120.3 (10)
C4—C5—C6	122.1 (2)	O5'—C16'—H16B	104.5
C4—C5—H5	118.9	C17'—C16'—H16B	104.5
C6—C5—H5	118.9	C15'—C16'—H16B	104.5
C5—C6—C1	118.9 (2)	C16—C17—N2	113.2 (9)
C5—C6—C7	117.7 (4)	C16—C17—H17A	108.9
C1—C6—C7	122.8 (4)	N2—C17—H17A	108.9
C5—C6—C7'	116.8 (5)	C16—C17—H17B	108.9
C1—C6—C7'	122.1 (5)	N2—C17—H17B	108.9
N1—C7—C6	127.7 (7)	H17A—C17—H17B	107.7
N1—C7—H7A	116.1	C16'—C17'—N2	119.4 (13)
C6—C7—H7A	116.1	C16'—C17'—H17C	107.5
N1'—C7'—C6	128.1 (10)	N2—C17'—H17C	107.5
N1'—C7'—H7B	115.9	C16'—C17'—H17D	107.5
C6—C7'—H7B	115.9	N2—C17'—H17D	107.5
O2—C8—C9	119.52 (19)	H17C—C17'—H17D	107.0
O2—C8—C13	122.0 (2)	H1W—O1W—H2W	114.4
C9—C8—C13	118.5 (2)	H3W—O2W—H4W	107.6
C10—C9—C8	120.6 (2)		
			
O1—Ni1—N1—C7	12.6 (5)	Ni1—N1—C7—C6	−2.5 (9)
N2—Ni1—N1—C7	−171.6 (5)	C5—C6—C7—N1	177.9 (5)
N1'—Ni1—N1—C7	−81.7 (9)	C1—C6—C7—N1	−11.2 (8)
O1—Ni1—N1—C15	−173.2 (4)	C7'—C6—C7—N1	84 (2)
N2—Ni1—N1—C15	2.6 (4)	C15'—N1'—C7'—C6	−179.1 (8)
N1'—Ni1—N1—C15	92.5 (8)	Ni1—N1'—C7'—C6	9.5 (13)
O2—Ni1—N1'—C7'	−81.6 (9)	C5—C6—C7'—N1'	171.9 (8)
O1—Ni1—N1'—C7'	−14.4 (7)	C1—C6—C7'—N1'	8.9 (12)
N2—Ni1—N1'—C7'	163.9 (7)	C7—C6—C7'—N1'	−90 (2)
N1—Ni1—N1'—C7'	71.9 (9)	Ni1—O2—C8—C9	−172.50 (17)
O2—Ni1—N1'—C15'	107.4 (7)	Ni1—O2—C8—C13	6.3 (3)
O1—Ni1—N1'—C15'	174.5 (5)	O2—C8—C9—C10	177.8 (2)
N2—Ni1—N1'—C15'	−7.2 (5)	C13—C8—C9—C10	−1.1 (4)
N1—Ni1—N1'—C15'	−99.1 (10)	C8—C9—C10—O4	−174.1 (3)
O2—Ni1—N2—C14	−0.8 (2)	C8—C9—C10—C11	3.7 (4)
N1—Ni1—N2—C14	−175.2 (3)	O4—C10—C11—C12	175.0 (3)
N1'—Ni1—N2—C14	162.4 (3)	C9—C10—C11—C12	−2.9 (5)
O2—Ni1—N2—C17'	170.2 (9)	C10—C11—C12—C13	−0.4 (5)
N1—Ni1—N2—C17'	−4.2 (9)	O2—C8—C13—C12	179.1 (2)
N1'—Ni1—N2—C17'	−26.6 (9)	C9—C8—C13—C12	−2.1 (4)
O2—Ni1—N2—C17	−169.7 (6)	O2—C8—C13—C14	−3.0 (4)
N1—Ni1—N2—C17	16.0 (6)	C9—C8—C13—C14	175.8 (2)
N1'—Ni1—N2—C17	−6.4 (7)	C11—C12—C13—C8	2.9 (4)
O2—Ni1—O1—C1	169.3 (2)	C11—C12—C13—C14	−175.2 (3)
N1—Ni1—O1—C1	−15.8 (3)	C17'—N2—C14—C13	−167.9 (8)
N1'—Ni1—O1—C1	6.5 (3)	C17—N2—C14—C13	174.3 (6)
O1—Ni1—O2—C8	172.1 (2)	Ni1—N2—C14—C13	3.8 (4)
N2—Ni1—O2—C8	−4.1 (2)	C8—C13—C14—N2	−2.3 (5)
N1'—Ni1—O2—C8	−118.9 (6)	C12—C13—C14—N2	175.6 (3)
Ni1—O1—C1—C2	−171.24 (17)	C7—N1—C15—C16	133.4 (6)
Ni1—O1—C1—C6	8.0 (4)	Ni1—N1—C15—C16	−41.3 (7)
O1—C1—C2—C3	178.5 (2)	C7'—N1'—C15'—C16'	−124.1 (9)
C6—C1—C2—C3	−0.8 (4)	Ni1—N1'—C15'—C16'	47.5 (8)
C1—C2—C3—O3	−176.0 (2)	N1—C15—C16—O5	−167.0 (19)
C1—C2—C3—C4	2.8 (4)	N1—C15—C16—C17	74.6 (9)
O3—C3—C4—C5	176.4 (2)	N1'—C15'—C16'—O5'	165 (2)
C2—C3—C4—C5	−2.4 (4)	N1'—C15'—C16'—C17'	−63.0 (13)
C3—C4—C5—C6	0.0 (4)	O5—C16—C17—N2	−172.5 (14)
C4—C5—C6—C1	2.0 (4)	C15—C16—C17—N2	−54.6 (11)
C4—C5—C6—C7	173.2 (4)	C14—N2—C17—C16	−163.7 (6)
C4—C5—C6—C7'	−161.6 (5)	C17'—N2—C17—C16	88 (4)
O1—C1—C6—C5	179.2 (2)	Ni1—N2—C17—C16	6.4 (11)
C2—C1—C6—C5	−1.5 (4)	O5'—C16'—C17'—N2	157 (2)
O1—C1—C6—C7	8.4 (5)	C15'—C16'—C17'—N2	27.0 (19)
C2—C1—C6—C7	−172.3 (4)	C14—N2—C17'—C16'	−167.9 (10)
O1—C1—C6—C7'	−18.2 (6)	C17—N2—C17'—C16'	−89 (4)
C2—C1—C6—C7'	161.1 (5)	Ni1—N2—C17'—C16'	20.5 (17)
C15—N1—C7—C6	−177.1 (6)		

### Hydrogen-bond geometry (Å, º)

**Table:**

*D*—H···*A*	*D*—H	H···*A*	*D*···*A*	*D*—H···*A*
O1*W*—H1*W*···O2	0.82	1.96	2.770 (2)	167
O1*W*—H1*W*···O1	0.82	2.53	3.087 (2)	127
O1*W*—H2*W*···O4^i^	0.82	2.18	2.951 (3)	158
O2*W*—H3*W*···O1^ii^	0.82	1.98	2.787 (3)	168
O2*W*—H4*W*···O3^iii^	0.82	2.04	2.837 (2)	165
O3—H3*O*···O1*W*^iv^	0.82	1.87	2.665 (3)	165
O4—H4*O*···O2*W*^v^	0.82	1.81	2.626 (3)	171
O5—H5*OA*···O1*W*^vi^	0.82	1.97	2.78 (3)	166
O5′—H5*OB*···O1*W*^vi^	0.82	2.28	3.03 (4)	151

Symmetry codes: (i) *x*−1, *y*, *z*; (ii) *x*, *y*, *z*−1; (iii) *x*+1, *y*, *z*−1; (iv) −*x*+1, −*y*+1, −*z*+2; (v) −*x*+2, −*y*+1, −*z*+1; (vi) −*x*+3/2, *y*−1/2, −*z*+3/2.
